# Optimizing parenteral nutrition to achieve an adequate weight gain according to the current guidelines in preterm infants with birth weight less than 1500 g: a prospective observational study

**DOI:** 10.1186/s12887-021-02782-1

**Published:** 2021-07-07

**Authors:** Nan Wang, Lianlian Cui, Zhen Liu, Yan Wang, Yuhua Zhang, Changsong Shi, Yanbo Cheng

**Affiliations:** 1 Department of Pediatric Gastroenterology, Hepatology and Nutrition, Henan Provincial People’s Hospital, People’s Hospital of Zhengzhou University, 7 Wei Wu Road, Zhengzhou, 450003 Henan China; 2 Department of Neonatology, Henan Provincial People’s Hospital, People’s Hospital of Zhengzhou University, Zhengzhou, 450003 Henan China; 3 Department of Pediatric Intensive Care Unit, Henan Provincial People’s Hospital, People’s Hospital of Zhengzhou University, Zhengzhou, 450003 Henan China

**Keywords:** Parenteral nutrition, Very low birth weight infants, Extremely low birth weight infants, Weight gain

## Abstract

**Aim:**

European Society for Clinical Nutrition and Metabolism released the guidelines on pediatric parenteral nutrition in 2018. We aimed to compare the parenteral nutrition (PN) regimen with the current guidelines, evaluate weight gain and explore the correlation of parenteral macronutrient and energy intakes with weight gain outcome in preterm infants with birth weight less than 1500 g.

**Methods:**

A prospective observational study was conducted. Parenteral macronutrients and energy intakes were described. Weight gain during PN was assessed. Nutritional factors associated with weight gain outcome after PN were identified using a cox proportional hazards model.

**Results:**

A total of 163 infants were included in this study, in which 41 were extremely low birth weight (ELBW) infants and 122 were very low birth weight (VLBW) infants. Average glucose, amino acid, lipid, and energy during the first postnatal week were 7.5 g/kg/d, 2.4 g/kg/d, 0.8 g/kg/d, 48 kcal/kg/d. Median maximum glucose, amino acid, lipid, and energy were 11.1 g/kg/d, 3.5 g/kg/d, 3 g/kg/d, 78 kcal/kg/d. Median days to maximum glucose, amino acid, lipid, and energy were 10, 9, 12, 11 days. The proportion of appropriate for gestational age (AGA) infants was 76.9%. The ratio of infants without poor weight gain outcome after PN was 38%. With every 0.1 g/kg/d decrease of maximum amino acid and average lipid during the first postnatal week, the probability of appropriate weight gain outcome decreased by 77.6 and 74.4% respectively. With each additional day to maximum glucose and energy, the probability of appropriate weight gain outcome decreased by 5.6 and 6.1% respectively.

**Conclusions:**

Most preterm infants with birth weight less than 1500 g remain below the latest recommended nutrition goals. The poor weight gain outcome of these infants after PN is related to insufficient parenteral macronutrient and energy intakes. PN strategies should be improved according to the latest evidence-based recommendations.

**Supplementary Information:**

The online version contains supplementary material available at 10.1186/s12887-021-02782-1.

## Background

Parenteral nutrition (PN) is critical for the care of preterm infants [1] and provides relatively safe means of meeting nutrient intakes [2], as their immature gastrointestinal (GI) tract cannot tolerate sufficient energy and nutrients enterally to meet nutrient requirements [3]. PN for preterm infants is a highly complex combination of amino acids, lipid emulsions, carbohydrates, electrolytes, vitamins, and minerals that differs significantly from the adult PN [1]. What is optimal PN for very preterm infants remains unanswered [4]. Several guidelines have been established to help improve the management of nutritional needs in preterm infants. Chinese Society of Parenteral and Enteral Nutrition (CSPEN) guidelines for nutrition support in neonates have been used in our center since 2013 [5]. In 2018, the European Society for Clinical Nutrition and Metabolism (ESPEN) released the guidelines on pediatric PN [6–9]. Subtle differences exist between the guidelines in terms of amino acids, lipid emulsions, glucose and energy intakes, and timings. Currently, the fact that nutrition practice differs from the recommendations established in the guidelines and the recommended intake is not administered in most cases is widespread [10, 11].

As clinical neonatologists have increasingly recognized that it may be inappropriate to compare preterm infant growth with the growth of their in utero counterparts, some new insights in preterm nutrition have been proposed [12]. A recent study found that improved nutritional supply and growth during early life have been related to the better long-term growth of brain structures and a more favorable neurodevelopmental outcome [13]. Another research revealed that very rapid catch-up growth after a period of growth faltering has been linked to adverse metabolic consequences [14]. So, “adequate” instead of “aggressive” nutrient supply should be provided to reach a positive weight gain and prevent postnatal growth faltering. To date, there is no international consensus regarding what constitutes the ideal pattern of growth of preterm infants [15]. Some authors suggested that the growth assessments should be comprehensive, including weight, length, and head circumference measures, and if possible, fat and fat-free mass [15, 16]. Weight gain is typically measured rather than overall growth anthropometric measures in current clinical practice [15].

We aimed to analyze the PN regimen, compare it with ESPEN guidelines on pediatric parenteral nutrition in 2018 [6–9], evaluate weight gain during PN, and explore the correlation of macronutrients and energy of PN with weight gain outcome after PN in VLBW and ELBW infants.

## Methods

### Study design and patients

This was a prospective observational single center cohort study. NICU (Zhengzhou, China) in this study is the neonatal critical care center in Henan Province, specializing in the care of ill or premature newborn infants. Additionally, the hospital is also a high-risk maternity center in Henan Province. Almost all the infants admitted to the NICU came from the obstetrics department in the hospital. Preterm infants, who were admitted to the NICU immediately after birth from July 20, 2018, to January 27, 2020, with gestational age (GA) < 37 weeks, birth weight (BW) < 1500 g were eligible for enrollment. The exclusion criterion was congenital metabolic diseases. No chromosomal abnormalities were identified in the study population. We also did not include infants who died during hospitalization or the treatment of those whom was withdrawn by a parent.

### Nutrition management

Parenteral nutrition: Individualized PN was prescribed daily. The PN solution containing amino acid (Pediatric Compound Amino Acid Injection 19AA-I, PAA 6%, Qidu, Shangdong, China), lipid (20% Medium and Long Chain Fat Emulsion Injection C6 ~ 24, Fresenius Kabi, Wuxi, Jiangsu, China), glucose, minerals, trace elements (Addamel; Fresenius Kabi, Wuxi, Jiangsu, China), water-soluble vitamins (Soluvit; Fresenius Kabi, Wuxi, Jiangsu, China) and fat-soluble vitamins (Vitalipid; Fresenius Kabi, Wuxi, Jiangsu, China) was started within the first 24 h of life and infused continuously for 24 h.

Enteral nutrition: Minimal enteral nutrition (MEN) was initiated as soon as possible after birth if applicable. A preterm formula (PreNan, Nestle, Germany) or mother’s own milk (MOM) was administrated through a nasogastric tube intermittently. Breastmilk energy estimates range from 65 to 70 kcal/dL [17]. We chose 67 kcal/dL to calculate the energy estimates.

### Data collection and management

Clinical information was collected prospectively, including gender, GA, BW, 5-min Apgar score, and late-onset sepsis. Bodyweight was measured daily by NICU nurses and converted to weight z-score based on the 2013 Fenton growth chart. The weight gain outcome was assessed on the next day of stopping PN. Poor weight gain outcome was defined as body weight below the 10th percentile for postmenstrual age, as plotted on the 2013 Fenton growth curves. Conversely, appropriate weight gain was defined as body weight above the 10th percentile for postmenstrual age. Extrauterine growth restriction (EUGR) is diagnosed when weight is <10th percentile at discharge [18]. Late-onset sepsis was defined as a positive culture from blood, cerebrospinal fluid, catheter, or suprapubic urine at ≥5 days of life. Nutrient intake information was collected during PN. Product monographs were used to determine the nutrient and energy composition of PN solutions and preterm formula. Estimated PN nutrient intakes were compared against the recommended intakes [6–9].

### Statistical analysis

Nutrient intake data were presented as median (first, third quartile). The difference of weight z-score/percentile before and after PN was tested using a paired t-test. The association between PN strategy and weight gain outcome was assessed using the cox proportional hazards model. Correlation analysis between clinical characteristics and factors associated with poor weight gain outcome after PN was assessed using Kendall’s tau-b coefficients. A *p*-value < 0.05 was considered statistically significant. Data were analyzed using SPSS 24.0 (SPSS, Inc., IBM, Chicago, IL, USA). We generated figures using GraphPad Prism V.7.0 (San Diego, CA, USA).

## Results

**Fig. 1:**
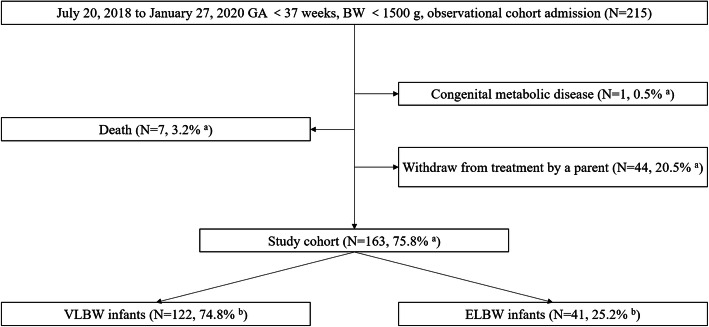
Flow chart of all preterm infants born with gestation age < 37 weeks, birth weight < 1500 g during the study period. a, the denominator is 215; b, the denominator is 163. GA, gestation age. BW, birth weight. VLBW, very low birth weight (defined as a birth weight of less than 1500 g). ELBW, extremely low birth weight (defined as a birth weight of less than 1000 g)

A total of 215 infants met the eligibility. During hospitalization, 7 died, 44 were withdrawn from treatment by a parent, and 1 had congenital metabolic diseases. The treatment of 44 infants was withdrawn by parents for reasons as follows: worry about a poor prognosis (19 infants); discharge against medical advice as clinical conditions improved (19 infants); transfer to a specialized hospital for congenital heart diseases (3 infants); inability to pay for treatment (3 infants). The remaining 163 infants were included, in which 41 were ELBW infants and 122 were VLBW infants (Fig. 1). Clinical characteristics were showed in Table 1. PN and EN support was summarized in Table 2.

**Table 1:** Clinical characteristics of the study sample (*n* = 163)

Characteristic	Value
Sex, n (%)	
Female	75 (46.0)
Male	88 (54.0)
Gestational age at birth, weeks	29.6 (28.3, 31.4)
Gestational age at parenteral nutrition discontinuation, weeks	34.3 (32.9, 36.0)
5-min Apgar score, n (%)	
≥ 7	154 (94.5)
< 7	9 (5.5)
Birth anthropometrics	
Birth weight, g	1150 (990, 1270)
Length, cm	34 (28, 38)
Head circumference, cm	28 (26, 36)
Small for gestational age, n (%)	39 (23.9)
Parenteral nutrition-associated cholestasis, n (%)	14 (8.6)
Sepsis, n (%)	14 (8.6)
Surgical conditions	
Necrotizing enterocolitis, n (%)	12 (7.4)
Duodenal atresia, n (%)	1 (0.6)
Congenital megacolon, n (%)	2 (1.2)
Patent ductus arteriosus, n (%)	3 (1.8)
Gastric perforation, n (%)	1 (0.6)
Others ^a^, n (%)	3 (1.8)
Ventilator, days	6 (1, 19)
Maximum weight loss, %	8.0 (5.0,11.0)
Days to maximum weight loss, days	5 (4, 6)
Poor weight gain outcome after parenteral nutrition, n (%)	101 (62.0)
Extrauterine growth restriction, n (%)	136 (83.4)

^a^ others including incarcerated inguinal hernia, anorectal fistula and urachal fistula. Poor weight gain outcome was defined as body weight below the 10th percentile for postmenstrual age, as plotted on the 2013 Fenton growth curves in this study. EUGR is diagnosed when weight is <10th percentile at discharge. Continuous variables are presented as median (interquartile)

**Table 2:** Characteristics of PN and EN support (*n* = 163)

Characteristic	Value
Duration of PN, days	30 (21, 40)
Average PN glucose during the first postnatal week, g/kg/d	7.5 (6.4, 8.5)
Average PN amino acid during the first postnatal week, g/kg/d	2.4 (2.1, 2.7)
Average PN lipid during the first postnatal week, g/kg/d	0.8 (0.4, 1.2)
Average PN energy during the first postnatal week, kcal/kg/d	48 (40, 52)
Maximum PN glucose, g/kg/d	11.2 (9.3, 13.0)
Maximum PN amino acid, g/kg/d	3.5 (3.2, 3.8)
Maximum PN lipid, g/kg/d	3.0 (2.6, 3.2)
Maximum PN energy, kcal/kg/d	78 (68, 88)
Days to reach maximum PN glucose, days	10 (6, 18)
Days to reach maximum PN amino acid, days	9 (6, 13)
Days to reach maximum PN lipid, days	12 (9, 17)
Days to reach maximum PN energy, days	11 (8, 17)
Days to start PN lipid, days	4 (2, 5)
PN amino acid, n (%)	
Day 1, > 1.5 g/kg/d	64 (39.3)
Day 2, > 2.5 g/kg/d	11 (6.7)
Day 7, > 2.5 g/kg/d	132 (81.0)
PN lipid, n (%)	
Day 1, yes	4 (2.5)
Day 2, yes	1 (0.6)
Day 7, yes	138 (84.7)
Day of life of first enteral feed, n (%)	
Day 1	27 (16.6)
Day 2	58 (35.6)
≥ Day 3	78 (47.8)
Days to reach full enteral feed, days	30 (23, 42)
EN energy when PN stopped, kcal/kg/d	108 (100, 115)
Episodes of EN interruption, n (%)	
0	104 (63.8)
1	36 (22.1)
2	12 (7.4)
3	9 (5.5)
4	2 (1.2)

*PN* parenteral nutrition, *EN* enteral nutrition. Continuous variables are presented as median (interquartile)

**Fig. 2:**
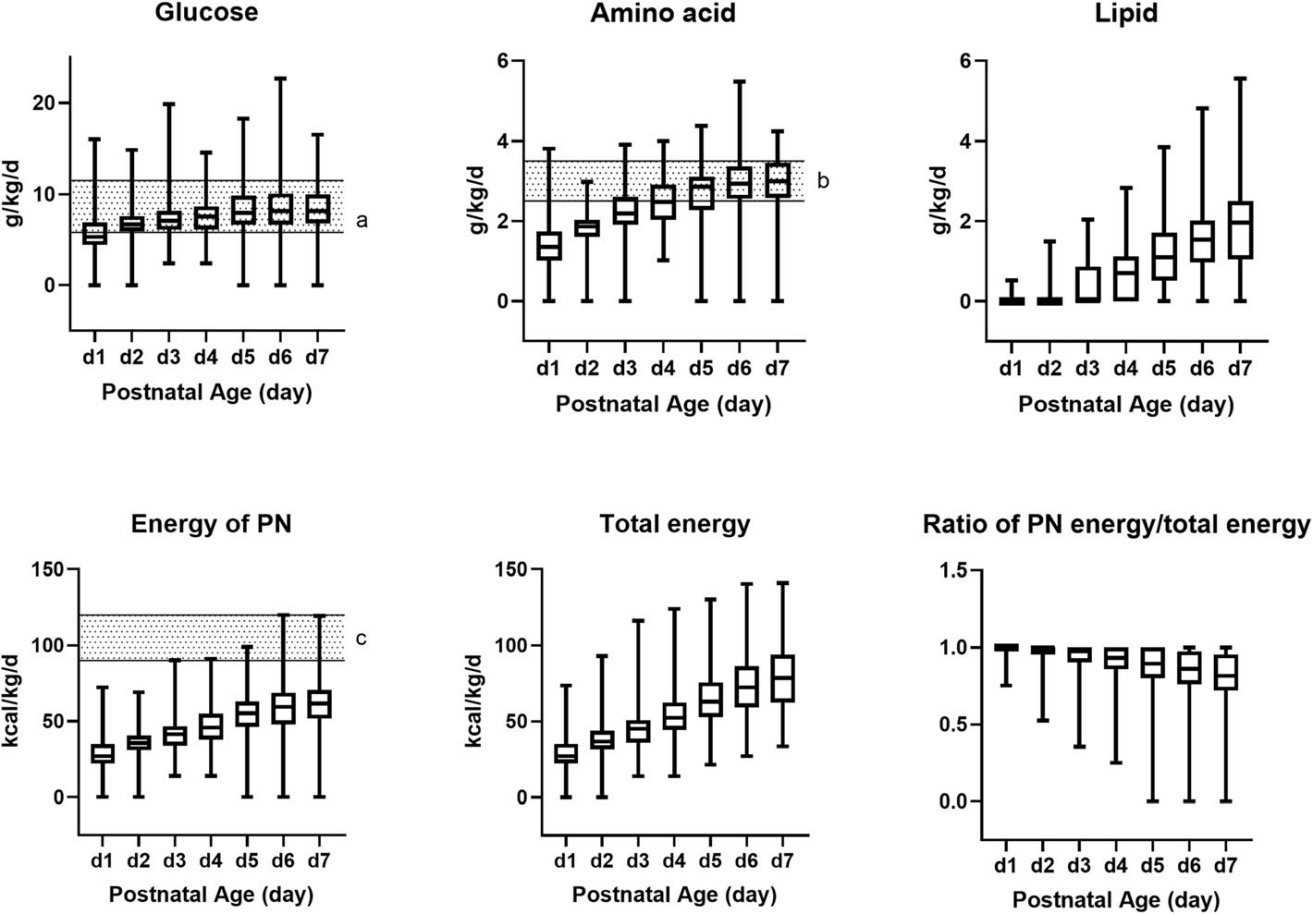
Daily macronutrient and energy intakes during the first postnatal week. PN, parenteral nutrition. Data are presented as median (first, third quartile). a, the recommended range of glucose intake on the first postnatal day [7]. b, the recommended range of amino acid intake from postnatal day 2 onwards [6]. c, the recommended range of PN energy intake provided in VLBW infants [9]

Amino acid started from 1.4 g/kg/d (day 1), then gradually increased to 3.0 g/kg/d (day 7). Though amino acid infusions were commenced in all infants on day 1, only 39.3% of infants received > 1.5 g/kg/d. On day 2, the proportion of infants receiving > 2.5 g/kg/d was 6.7%. By day 7, 19.0% of infants still did not receive 2.5 g/kg/d amino acid. Most infants received lipid infusion on day 4 or even later. The median of lipid intake was 0.7 g/kg/d (day 4), and increased slowly to 2.0 g/kg/d (day 7). Only 4 (2.5%) infants received lipid on day 1. All infants did not reach the recommended glucose intake of 5.8 g/kg/d on day 1, and the median glucose intake was 5.3 g/kg/d. All infants had an increase of glucose intake from 6.7 g/kg/d (day 2) to 8.2 g/kg/d (day 7). The intakes also did not approach the recommended 11.5 g/kg/d [7]. Median PN energy increased gradually from 27 kcal/kg/d on day 1 to 62 kcal/kg/d on day 7, the median proportion of which accounted for 100 to 80% in total energy. PN makes a significant contribution toward meeting the nutrient goals in the first week. (Fig. 2, Supplementary Table 1, Table 2).

**Fig. 3:**
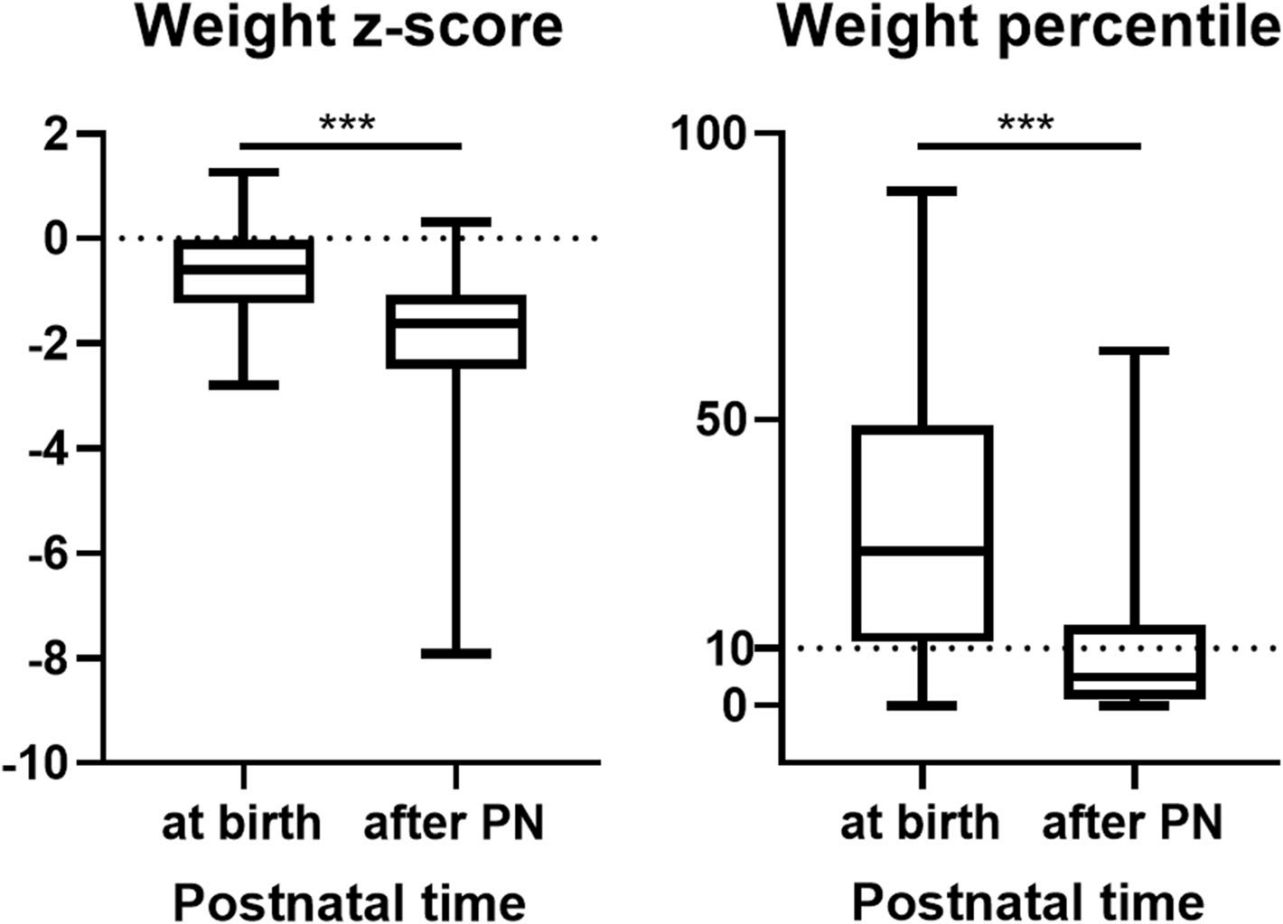
Changes of weight z score and weight percentile at birth and after PN. PN, parenteral nutrition. Data are presented as median (first, third quartile). ^***^ *P* < 0.001

The weight changes of infants were demonstrated in Fig. 3. Median weight z score at birth was − 0.61, then with a persisting declination during PN, decreased significantly to − 1.63. Similarly, the median weight percentile decreased from 27 to 5%. The proportion of small for gestational age (SGA) infants was 23.1%. Poor weight gain outcome after PN was observed in 62% of infants.

Average glucose/amino acid/lipid during the first postnatal week, maximum glucose/amino acid/lipid/PN energy, Days to reach maximum glucose/amino acid/lipid/PN energy, and days to start PN lipid were entered into separate cox proportional hazards ratio models to examine their association with the likelihood of poor weight gain outcome after PN (Table 3). We found that with every 0.1 g/kg/d decrease of average lipid during the first postnatal week and maximum amino acid, the probability of appropriate weight gain outcome decreased by 77.6 and 74.4% respectively. With each additional day to maximum glucose and energy, the probability of appropriate weight gain outcome decreased by 5.6 and 6.1% respectively. We then conducted a correlation analysis between clinical characteristics and the factors of PN affecting weight gain outcome (Table 4). GA, BW was negatively correlated with maximum amino acid, time to reach maximum glucose, and PN energy. Surgery and duration of ventilator were positively correlated with maximum amino acid, time to reach maximum glucose, and PN energy. Sepsis was positively correlated with the time to reach maximum glucose.

**Table 3:** Association between PN strategy and weight gain outcome in cox regression models

PN strategy	*P* value	Exp(B)	95%CI for exp.(B)	
			lower	upper
Average glucose during the first postnatal week	.329	0.869	.656	1.151
Average amino acid during the first postnatal week	.410	1.562	.540	4.520
Average lipid during the first postnatal week	.016	.256	.085	.772
Maximum glucose, g/kg	.705	.963	.795	1.168
Maximum amino acid, g/kg	.000	.224	.102	.493
Maximum lipid, g/kg	.250	1.437	.775	2.667
Maximum PN energy, kcal/kg	.312	1.262	.804	1.982
Days to reach maximum glucose, days	.008	.944	.904	.985
Days to reach maximum amino acid, days	.092	.959	.914	1.007
Days to reach maximum lipid, days	.321	.975	.927	1.025
Days to reach maximum PN energy, days	.041	.939	.884	.998
Days to start PN lipid, days	.128	.822	.638	1.058

*PN* parenteral nutrition, *CI* confidence interval

**Table 4:** Correlation analysis between clinical characteristics and factors associated with weight gain outcome after PN

Clinical characteristics	Factors associated with weight gain outcome after PN			
	Maximum amino acid	Days to reach maximum glucose	Days to reach maximum PN energy	Average lipid ^a^
Gender	.017 (.797)	−.032 (.624)	.074 (.260)	−.075 (.259)
GA	−.187 (.001)	−.178 (.001)	−.207 (.000)	−.033 (.542)
5-min Apgar score	.091 (.169)	.096 (.145)	.116 (.078)	−.114 (.085)
BW	−.236 (.000)	−.223 (.000)	−.239 (.000)	.000 (.995)
SGA	.093 (.162)	.083 (.205)	.103 (.118)	−.023 (.724)
PNAC	.078 (.238)	.113 (.085)	.112 (.088)	.005 (.941)
Sepsis	.098 (.139)	.149 (.023)	.102 (.119)	.025 (.706)
Surgical conditions	.201 (.002)	.259 (.000)	.227 (.001)	.021 (.750)
Ventilator	.205 (.000)	.297 (.000)	.326 (.000)	−.104 (.063)

^a^ Average lipid intake during the first postnatal week. *PN* parenteral nutrition, *GA* gestational age, *BW* birth weight, *SGA* small for gestational age, *PNAC* parenteral nutrition-associated cholestasis. Values are reported as Kendall’s tau b correlation coefficient (*p*-value)

## Discussion

In recent years, much attention has been focused on enhancing the nutritional support of very low birth weight infants [19]. Despite international guidance, PN strategy varies widely in clinical practice [20, 21]. In this study, the PN regimen used was not adequate according to the latest guideline on the whole [6–9].

Amino acid: in this study, many infants did not receive the starting dose of at least 1.5 g/kg/d on the first postnatal day. Days to reach maximum PN amino acid [9 (6, 13) days] were longer than the recommendation [6]. And, the maximum amino acid intake was 3.5 (3.2, 3.8) g/kg/d. Clinicians were still not used to initiating PN amino acid at a higher level despite evidence from studies demonstrating the safety of providing higher levels of amino acids as early as the first day of postnatal life [22–25]. A secondary analysis revealed that ELBW infants who received a minimum of 3 g/kg/d of parenteral amino acid in the first 5 days of life were found to have significantly better growth outcomes [26], suggesting that ELBW infants can tolerate the amino acid dose of 3–3.5 g/kg/d well within the first few days of life. A study evaluating metabolic responses to early high amino acid supplementation found serum blood urea nitrogen was higher when the infants were provided a higher dose of amino acid [27], which reflects a suboptimal balance of amino acids for unstable or “sick” preterm infants [28]. Also, recent data does not suggest improvement in growth or neurodevelopmental outcomes with use of parenteral amino acid greater than 3.5 g/kg/day in premature infants [29]. We should increase the starting dose of parenteral amino acid to 1.5 g/kg/d at least, decrease the maximum amino acid intake and shorten the days to reach maximum PN amino acid to improve the weight gain outcome of our infants. Further study should be implemented.

Lipid: our data suggest that lipid, the initiation of which was late (postnatal day 4 or later), was not an integral part of PN in the first few days. In other words, the PN solution was not an ‘all-in-One’ solution. Maximum lipid intake [3.0 (2.6, 3.2) g/kg/d] was lower than the previous studies [11, 30, 31]. Days to reach maximum PN lipid [12 (9, 17) days] were also long. There has been a trend toward earlier and higher rates of infusion of lipid for preterm infants [28]. A recent study revealed that provision of a high early dose of parenteral lipid in the first week of age (started on 2 g/kg/d within postnatal 24 h and increased to 3 g/kg/d the following day) results in less weight loss and lower incidence of EUGR. Lipid emulsions are an indispensable part of PN as a non-carbohydrate source of energy and the intake of 25–50% non-protein calories is recommended in fully parenterally fed infants [9, 11]. Lipid plays an important role in brain development since it represents a large percentage of the brain’s composition [32]. Lipid intake in our study, initiating late and providing with a relatively low dosage, obviously leads to compromising the provision of energy and may cause a deficit of essential fatty acids, which was not investigated here. Anyhow, earlier and faster provision of parenteral lipid should be carried out in the future.

Glucose: the glucose provision was also relatively conservative. Though all infants received the dose of glucose during the first week of life within the recommendation basically, median maximum intake was less than 11.5 g/kg/d [7]. Clinicians adopted this strategy to avoid overfeeding, which may be responsible for hyperglycemia, causes increased lipogenesis and fat tissue deposition together with subsequent liver steatosis, and so on [7]. No infants in our center showed hyperglycemia (data not shown). Glucose is the main energy source and the most widely used carbohydrate in total parenteral nutrition [33]. This strategy may also lead to compromising the provision of energy.

Energy: PN energy accounting for most of the total energy during the first postnatal week ranged from 27 kcal/kg/d to 62 kcal/kg/d. Besides, maximum PN energy was 78 kcal/kg/d. Both were much lower than the recommendations [9]. Days to reach maximum PN energy [11 (8, 17) days] were long, suggesting a slow increase. In the provision of parenteral amino acid, protein-to-energy ratios are important, most authorities suggest 25–40 kcal of non-protein energy is required per 1 g amino acid to promote lean mass accretion [34]. The energy provided in this study obviously cannot guarantee the rational use of amino acids. More importantly, adequate nutrition is critical to prevent early postnatal growth retardation and to optimize long-term growth and development [35]. Inadequate PN energy intake can be explained by the following reasons: lipid initiation was late and the dosage was relatively low; glucose intake was also relatively low; the increase of lipid and glucose was slow. More adequate provision of lipid and glucose should be attached importance to.

Growth assessment: In this study, 23.9% of infants were SGA at birth. Poor weight gain outcome after PN was observed in 62% of infants. More than half of premature infants suffered poor weight gain during PN. Though avoidance of excessive rates of early growth could have a role in the prevention of cardiovascular disease [36], there are no data to suggest an overall benefit of limiting nutrient intake, or restricting growth in preterm infants [37]. Meanwhile, strong data suggest that inadequate nutrition and growth may permanently affect brain outcomes [38, 39]. So, we should do our best to optimize the early weight gain of preterm infants currently.

Optimizing PN regimen: in this study, average lipid during the first postnatal week, maximum amino acid, and days to reach maximum glucose / PN energy were all associated with weight gain outcome after PN. Nutrient intakes were independent predictors of growth [40]. Although no large randomized controlled studies have been reported so far, early and moderate provision of lipid is supported by evidence. A retrospective study on 121 ELBW infants reports a negative correlation between the delay in introducing lipid emulsions and growth in the 1st month in preterm PN [41]. Another research revealed that neurodevelopment assessed at 1 year of age correlated with cumulative fat intake in the first 14 days [42]. Also, lipid supply, which decreases glucose utilization [43], is crucial for the maintenance of blood glucose in preterm infants [4]. Infants who received higher protein intakes (3.8 g/kg/d) had a significantly greater growth velocity over the first 30 days after regaining birth weight and a lesser z-score change between birth and discharge for weight [44]. However, ELBW infants (4 g/kg/d amino acid group) received an extra 8 g/kg of amino acids over the first 10 days of life without any significant difference in short- and long-term growth [45]. Maximum PN amino acid in this study have reached the recommendation of 3.5 g/kg/d [6]. Increase of maximum PN amino acid needs further investigation. Though brain accounts for 90% of whole-body glucose utilization in the neonate [46], parenteral glucose supply should be guided by the balance between meeting energy needs, the risk of overfeeding/excess glucose load, phase of illness, parenteral amino acid, parenteral lipid, etc. with medication [7]. It is unreasonable to aim for the target amount as soon as possible. Lipid and glucose contribute to the energy of parenteral nutrition. The primary shortcoming in parenteral energy supply in our clinical practice is low late lipid supply and relatively low glucose supply. There is no evidence that gradual increments in the infusion rate of lipids improve fat tolerance [8]. We should provide parenteral lipid earlier and higher to improve the energy supply.

We analyzed some clinical factors that may affect the behavior of prescribing parenteral nutrition regimen. The maximum amino acid, days to reach maximum either glucose or PN energy were negatively correlated with GA and BW. We failed to identify the factors affecting average lipid during the first postnatal week in the statistic model. Therefore, a more adequate parenteral nutrition strategy should be implemented to meet the evidence-based recommended intakes, further to optimize the weight gain regardless of GA, BW.

## Conclusions

PN strategy affects the growth of VLBW and ELBW infants. The parenteral nutrition strategy used in our center was not reasonable according to the latest recommendations, a more adequate PN regimen, especially parenteral lipid, should be implemented to meet the evidence-based recommended intakes, further to optimize the weight gain of preterm infants with BW less than 1500 g.

## Supplementary Information

**Additional file 1.**

## Data Availability

The datasets used and/or analyzed during the current study are available from the corresponding author on reasonable request.

## Abbreviations

AGA: Appropriate for gestational age
BW: Birth weight
CSPEN: Chinese Society of Parenteral and Enteral Nutrition
ELBW: Extremely low birth weight
EN: Enteral nutrition
ESPEN: European Society for Clinical Nutrition and Metabolism
EUGR: Extrauterine growth restriction
GA: Gestational age
GF: Growth failure
GV: Growth velocity
MEN: Minimal enteral nutrition
MOM: Mother’s own milk
NEC: Necrotizing enterocolitis
NICU: Neonatal intensive care units
PDA: Patent ductus arteriosus
PN: Parenteral nutrition
PNAC: Parenteral nutrition-associated cholestasis
SGA: Small for gestational age
VLBW: Very low birth weight
