# Supplementary material for: Optimizing parenteral nutrition to achieve an adequate weight gain according to the current guidelines in preterm infants with birth weight less than 1500 g: a prospective observational study
Source: BMC Pediatr. 2021 Jul 7;21:303. doi: 10.1186/s12887-021-02782-1 (PMC8260575; doi:10.1186/s12887-021-02782-1)
Supplement: Supplementary file 1 — Additional file 1. [file 12887_2021_2782_MOESM1_ESM.docx]

**Optimizing Parenteral Nutrition to Achieve an Adequate Weight Gain According to the Current Guidelines in Preterm Infants with Birth Weight Less than 1500g: A Prospective Observational Study**

Nan Wang^1^, Lianlian Cui^1^, Zhen Liu^2^, Yan Wang^1^, Yuhua Zhang^1^, Changsong Shi^3^, Yanbo Cheng^1*^

^1^Department of Pediatric Gastroenterology, Hepatology and Nutrition, Henan Provincial People’s Hospital, People’s Hospital of Zhengzhou University, Zhengzhou, Henan, 450003, China

^2^Department of Neonatology, Henan Provincial People’s Hospital, People’s Hospital of Zhengzhou University, Zhengzhou, Henan, 450003, China

^3^Department of Pediatric Intensive Care Unit, Henan Provincial People’s Hospital, People’s Hospital of Zhengzhou University, Zhengzhou, Henan, 450003, China

*Corresponding author

Yanbo Cheng

Department of Pediatric Gastroenterology, Hepatology and Nutrition

Henan Provincial People’s Hospital, People’s Hospital of Zhengzhou University

7 Wei Wu Road, 450003, Zhengzhou, Henan, China

Phone: +86 (0371) 65580775

E-Mail: [13838562435@163.com](mailto:13838562435@163.com)

Supplementary Table 1. Daily macronutrient and energy intakes during the first postnatal week (n = 163).

|  | Glucose  g/kg/d | Amino acid  g/kg/d | Lipid  g/kg/d | PN energy  kcal/kg/d | Total energy  kcal/kg/d | PN energy/total energy |
| --- | --- | --- | --- | --- | --- | --- |
| Day 1 | 5.3 (4.4-6.9) | 1.4 (1.0-1.8) | 0.0 (0.0-0.0) | 27 (22-35) | 27 (22-35) | 1.0 (1.0-1.0) |
| Day 2 | 6.7 (5.9-7.6) | 1.9 (1.6-2.0) | 0.0 (0.0-0.1) | 36 (31-41) | 37 (32-44) | 1.0 (1.0-1.0) |
| Day 3 | 7.1 (6.1-8.2) | 2.2 (1.9-2.6) | 0.0 (0.0-0.9) | 41 (34-47) | 45 (36-51) | 1.0 (0.9-1.0) |
| Day 4 | 7.5 (6.1-8.7) | 2.5 (2.0-2.9) | 0.7 (0.0-1.1) | 46 (38-55) | 52 (44-62) | 0.9 (0.9-1.0) |
| Day 5 | 7.9 (6.6-9.9) | 2.9 (2.3-3.1) | 1.1 (0.5-1.7) | 55 (46-63) | 63 (53-76) | 0.9 (0.8-1.0) |
| Day 6 | 8.2 (6.7-10.1) | 2.9 (2.6-3.4) | 1.5 (1.0-2.0) | 59 (48-69) | 72 (59-86) | 0.9 (0.8-1.0) |
| Day 7 | 8.2 (6.8-10.0) | 3.0 (2.6-3.5) | 2.0 (1.0-2.5) | 62 (52-71) | 78 (62-94) | 0.8 (0.7-1.0) |

Continuous variables are presented as median (interquartile).
